# Student Behavior Recognition in the Classroom Based on Hyper-YOLO

**DOI:** 10.3390/s26175601

**Published:** 2026-09-03

**Authors:** Jintao Sun, Jian Wang, Ming Yu

**Affiliations:** 1 College of Control Engineering, Northeast Forestry University, Harbin 150040, China; shenyi@nefu.edu.cn; 2 College of Computer and Artificial Intelligence, Northeast Forestry University, Harbin 150040, China; yuming@nefu.edu.cn

**Keywords:** classroom student behavior recognition, object detection, Hyper-YOLO, attention mechanism, feature pyramid, loss function

## Abstract

Automatic classroom student behavior recognition faces three major challenges: the coexistence of small targets and complex backgrounds, the conflict between details and semantics, and the interference from low-quality samples. To address these issues, this paper proposes a scene-driven integrated optimization framework, termed Hyper-YOLO-G. In the backbone network, a Squeeze-and-Excitation (SE) attention module is embedded to purify features and thereby enhance the channels of key behaviors. In the neck, a bidirectional weighted feature pyramid network (BiFPN) is introduced to perform scale-balanced multi-scale fusion. Moreover, the Wise-IoU v3 (WIoU v3) loss function is adopted to calibrate gradients and reduce the negative impact of low-quality samples. Experimental results on a public classroom behavior dataset show that Hyper-YOLO-G achieves 73.7% mAP@50 (mean average precision at IoU threshold 0.5), which is 4.9% higher than the baseline Hyper-YOLO. Ablation studies and generalization experiments on an independent multi-class dataset further demonstrate the combined effectiveness of each module and the generalization ability of the framework. This study provides a reliable technical path for high-precision, lightweight behavior recognition in complex classroom environments.

## 1. Introduction

In recent years, computer vision has significantly promoted the digital transformation of education. Building smart classrooms capable of automatically perceiving and analyzing the teaching process has become an important approach to improving educational quality [1,2,3]. Among these efforts, automated classroom behavior recognition plays a crucial role in objectively evaluating teaching interactions and student engagement. However, traditional manual observation is subjective and labor-intensive, while the complex classroom environment remains a major challenge for existing automated methods.

These challenges boil down to three core contradictions. First, the coexistence of small targets and large backgrounds: back-row hand-raising and subtle writing movements occupy only a small image proportion, are often occluded, and thus offer limited discriminative information. Second, the inconsistency between fine details and high-level semantics: classroom scenes contain both large-scale postures and subtle behavioral cues, requiring effective multi-scale feature representation. Third, the coexistence of high- and low-quality samples: occlusion, motion blur, and viewpoint variations produce numerous low-quality samples that interfere with stable optimization during training.

To address these challenges, deep learning-based object detection methods, especially one-stage YOLO detectors, have been widely adopted for their high efficiency [4]. The recently proposed Hyper-YOLO model introduced hypergraph computation to model high-order semantic associations [5], offering a new perspective on spatial and interaction relationships among students. However, Hyper-YOLO’s feature representation and optimization strategy are not tailored to classroom behavior detection. First, feature extraction lacks attention-based selection for key behavioral cues, making it difficult to focus on small targets against large backgrounds. Second, feature fusion relies on fixed concatenation and cannot adaptively weigh multi-scale contributions, failing to reconcile the detail-semantic contradiction. Third, localization optimization treats all samples equally and cannot suppress harmful gradients from low-quality samples, reflecting the contradiction between high-quality and low-quality samples.

Therefore, this paper proposes an integrated enhancement framework to address the above challenges. At the feature extraction stage, a Squeeze-and-Excitation (SE) attention mechanism [6] is introduced to enhance behavior-related channel responses while suppressing background interference. At the feature fusion stage, a bidirectional weighted feature pyramid network (BiFPN) [7] is employed to adaptively aggregate multi-scale features. During optimization, the Wise-IoU v3 (WIoU v3) loss [8] is adopted to alleviate the influence of low-quality samples through dynamic gradient adjustment. These three components are jointly integrated into an optimization framework consisting of feature enhancement, adaptive feature fusion, and robust localization optimization.

Building upon Hyper-YOLO, this work develops Hyper-YOLO-G, a scene-oriented integrated framework that combines lightweight channel attention, adaptive multi-scale feature fusion, and robust localization optimization. The main contributions are summarized as follows:

1. A lightweight channel enhancement strategy via SE module embedding to improve behavior-related feature representation while suppressing background interference.
2. An adaptive multi-scale feature fusion strategy based on BiFPN is introduced to improve feature interaction across different scales for classroom behavior detection.
3. A sample-quality-aware localization optimization strategy based on WIoU v3 is adopted to improve robustness against occlusion and blur.
4. The proposed framework effectively integrates the above three components, yielding consistent improvements on public classroom behavior datasets.

## 2. Related Work

This chapter aims to trace the evolution of object detection technologies through the lens of the core challenges faced by classroom behavior recognition. We focus on the limitations of existing methods in addressing the three contradictions—“small targets vs. large background”, “details vs. semantics”, and “high-quality vs. low-quality samples”—and critically analyze the deficiencies of current state-of-the-art models in constructing an integrated “perception-fusion-optimization” chain, thereby clearly positioning the motivation and innovative value of this work.

### 2.1. Classroom Behavior Recognition Based on Object Detection

Classroom behavior recognition is essentially a fine-grained object detection task applied to educational scenarios. Early studies mostly relied on two-stage detectors (e.g., Faster R-CNN) [9]. Although required for classroom analytics, their computationally heavy pipelines hinder real-time analysis and large-scale deployment. Subsequently, one-stage detectors represented by YOLO and SSD [10,11] became the mainstream choice due to their favorable balance between efficiency and accuracy. In recent years, researchers have customized detectors for classroom-specific challenges. For example, Cao et al. [12] proposed the MFD-YOLO algorithm based on YOLO11, which significantly improves the detection of small targets and dense student groups by designing a multi-dimensional feature flow network (MFFN) and a feature enhancement aggregation module (FEAM). Lin Zhang et al. [13] addressed the problem of low recognition accuracy caused by high target density and low image resolution in student classroom behavior recognition by integrating the SPD-Conv module [14] into YOLO11, enhancing the model’s ability to handle low-resolution images and small targets.

### 2.2. Detector Evolution and the Insights and Limitations of Hyper-YOLO

The YOLO series reflects a continuous pursuit of accuracy-efficiency balance, with innovations concentrated on the backbone (e.g., CSPDarknet), neck (e.g., PANet), and multi-scale prediction mechanisms [15]. The proposal of Hyper-YOLO marks an important paradigm shift: by introducing hypergraph computation, it makes the first explicit attempt to model complex high-order semantic and spatial associations among objects [16]. This is insightful for understanding non-local semantic relationships such as student interactions, offering a novel perspective to resolve the detail-vs-semantics contradiction at a high-order level.

However, while Hyper-YOLO pursues high-order modeling, its underlying basic capabilities are significantly misaligned with the front-end contradictions of classroom scenes. To address Hyper-YOLO’s decreased sensitivity to high-frequency features in complex environments, some studies have embedded an efficient channel attention (ECA) module into MANet (Mixed Aggregation Network) and adopted the EIoU loss for bounding box regression [17]. Although these improvements have achieved results in tasks such as coal mine foreign object detection, they still focus on single-module optimization and lack a systematic integrated design that spans feature extraction, fusion, and loss function. Therefore, the core premise of this work is that the basic perception and fusion capabilities must first be strengthened before the advantages of high-order semantic modeling can be fully exploited. Using Hyper-YOLO as a baseline, this work aims to perform a “systematic augmentation” by injecting targeted modules, thereby constructing a complete system from robust low-level perception to high-level semantic understanding.

### 2.3. Feature Enhancement and Fusion: From Mechanism Introduction to Integrated Design

To improve basic perception capabilities, attention mechanisms have been widely introduced into object detection. The Squeeze-and-Excitation (SE) module, as a classic channel attention mechanism, enhances the network’s response to important features by recalibrating channel weights. Su et al. [18] validated the effectiveness of combining the SE module with BiFPN and WIoU in pedestrian detection in complex scenes, showing that channel attention can effectively focus on key features. Regarding feature fusion, BiFPN achieves adaptive multi-scale fusion via cross-scale connections and learnable weights, effectively preserving small-target features in smart agriculture pest detection [19].

However, existing studies often treat feature enhancement and feature fusion as separate independent improvements [20]. This paper argues that, in the classroom context, screening out key features and effectively fusing them across multiple scales must be a closely integrated design process. Isolated enhancement of features or isolated improvement of fusion cannot fundamentally resolve the relational contradiction between “small targets” and “multi-scale”. Therefore, the proposed scheme deeply integrates SE’s channel selection with BiFPN’s weighted fusion, forming an integrated optimization pipeline from feature selection to multi-scale propagation and ensuring that enhanced key features are optimally integrated across scales.

### 2.4. Bounding Box Regression Loss: From Geometric Consistency to Quality Awareness

The accuracy of bounding box regression directly affects the usability of behavior localization. The evolution from Smooth L1 to IoU-series losses (GIoU, DIoU, CIoU) reflects a shift from coordinate regression toward geometric metric consistency [21]. However, homogeneous optimization strategies such as CIoU can harm model robustness by producing harmful gradients when faced with numerous low-quality samples caused by occlusion and blur.

The Wise-IoU (WIoU) series, through a dynamic non-monotonic focusing mechanism, innovatively introduces “sample quality” as a regulating factor for gradient allocation. Its core idea is to evaluate the outlier degree of anchor boxes and dynamically assign gradient gains, automatically suppressing the negative influence of low-quality outlier samples while preventing simple samples from dominating the training. Dong et al. validated the effectiveness of Wise-IoU in DMSA-Net for classroom behavior recognition scenarios, showing that the introduction of the dynamic non-monotonic focusing mechanism significantly improves model localization accuracy [22].

This paper introduces WIoU v3 as a key component to resolve the contradiction between “high-quality and low-quality samples” and to achieve “gradient calibration”. Together with the SE module (feature purification) and BiFPN (scale balancing), it forms an end-to-end integrated framework that spans input feature processing, multi-scale fusion, and final loss optimization, systematically addressing the three core challenges of classroom behavior recognition.

## 3. Methods

### 3.1. Workflow of the Proposed Model

**Figure 1:**
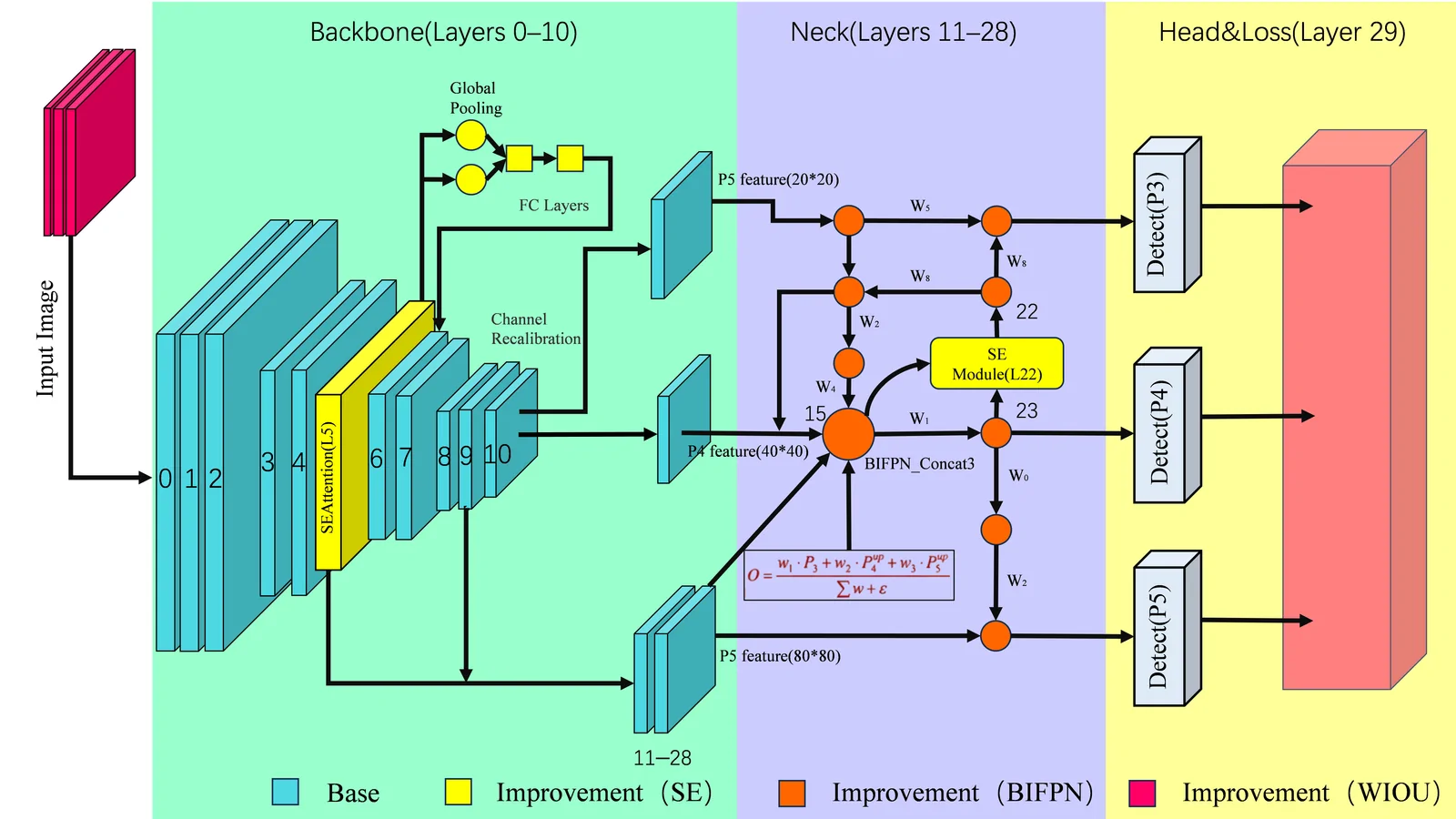
Neural network diagram of the improved model.

To systematically address the three core challenges in classroom behavior recognition—namely, weak key signals, large target scale variations, and uneven sample quality—and the three underlying contradictions (“small targets vs. large background”, “details vs. semantics”, “high-quality vs. low-quality samples”), this paper proposes a scene-driven progressive integrated optimization framework. Built upon Hyper-YOLO, the framework follows a progressive design philosophy of “feature purification → scale balancing → gradient calibration”, and systematically enhances the entire pipeline from feature extraction and fusion to loss optimization. The overall neural network architecture of the improved model is shown in Figure 1.

The workflow of the improved model is as follows. First, the input image passes through an enhanced backbone network integrated with the Squeeze-and-Excitation (SE) attention module to achieve feature purification, which suppresses complex background noise and adaptively focuses on key behavioral channels, directly addressing the “small targets vs. large background” contradiction. Subsequently, the extracted multi-scale features are fed into a bidirectional weighted feature pyramid network (BiFPN) for scale-balanced fusion using learnable weights, effectively reconciling the conflict between shallow details and deep semantics. Finally, during the training phase, the WIoU v3 loss function is introduced to perform gradient calibration through its dynamic non-monotonic focusing mechanism, automatically reducing the harmful influence of low-quality samples and improving the localization robustness under complex conditions. While maintaining the high efficiency of a one-stage detector, this framework constructs an end-to-end optimization solution tailored to classroom scenes through the three-level integrated optimization strategy.

Structural optimization strategy: In addition to embedding modules such as SE, BiFPN, and WIoU v3, the head structure of the original Hyper-YOLO is also simplified. In the original model, the semantic collection stage fuses features from five scales (B1 to B5). However, in the classroom behavior recognition scenario, we observe that the deeper feature layers (e.g., B1 and B2) contain limited detailed information while incurring relatively high computational costs. Therefore, in the improved model, the input to the semantic collection is simplified to three key scales (P3, P4, and P5), and the number of MANet modules in the subsequent fusion path is reduced accordingly. This optimization significantly reduces the model’s parameter count and computational complexity without sacrificing performance, providing a better foundation for edge deployment. We refer to this simplified backbone-and-neck structure as Hyper-YOLO-Light, which serves as the baseline for subsequent incremental integration of SE, BiFPN, and WIoU v3.

### 3.2. Algorithm Structure of Hyper-YOLO

**Figure 2:**
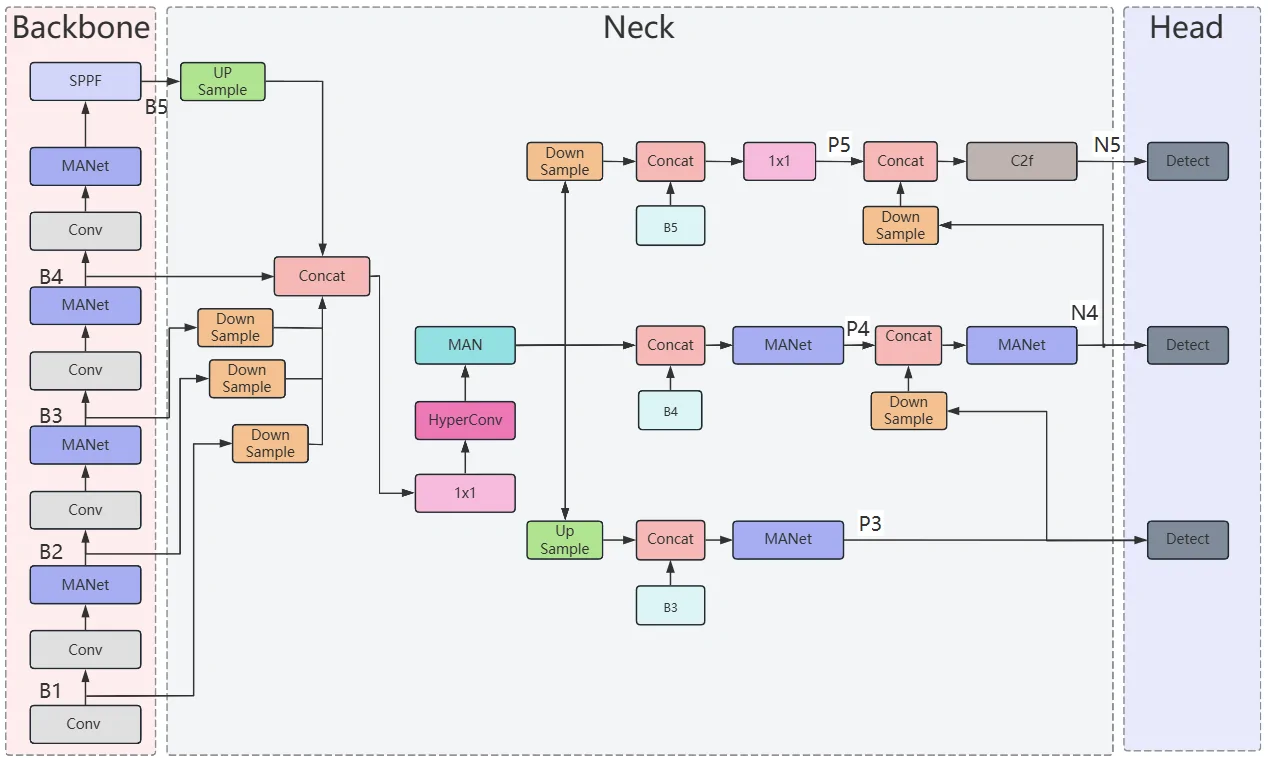
Hyper-YOLO Neural Network Diagram.

In this paper, the lightweight N-series version of Hyper-YOLO is adopted as the baseline model. Its core innovation lies in introducing hypergraph computation into object detection to model high-order semantic and spatial associations among feature points. However, in the classroom scenario, it is not specifically designed for enhancing small-target features, initializing multi-scale fusion weights, or optimizing localization under low-quality samples. As shown in the original configuration, its structure consists of a backbone network and a neck network. The specific neural network structure is shown in Figure 2.

Backbone network: It is stacked with standard convolutional layers and MANet modules, responsible for basic feature extraction. MANet integrates 1 × 1 convolution, depthwise separable convolution, and the C2f structure to achieve rich gradient flow and hierarchical feature integration.

Neck network: Its core pipeline comprises semantic collection → hypergraph computation → semantic scattering. First, feature maps from different levels of the backbone (B1 to B5) are aggregated via up-sampling and pooling operations with simple concatenation to form a mixed feature set. Then, this feature bag undergoes hypergraph construction and convolution operations to model high-order relationships among feature points in the semantic space. Finally, the enhanced semantic information is distributed back to output feature maps of different scales through a scattering mechanism for detection.

Although the high-order modeling capability of Hyper-YOLO provides a new perspective for understanding classroom interaction relationships, evident bottlenecks exist in its basic feature processing. First, feature extraction lacks targeted attention to key channels. Second, multi-scale fusion relies on simple equal-weight concatenation, limiting the efficiency of information fusion. This work is precisely designed to strengthen these fundamental bottlenecks so that high-order semantic modeling can be built on a more solid foundation.

### 3.3. Improvement Scheme for Hyper-YOLO

#### 3.3.1. Feature Extraction Enhancement in the Backbone Network

**Figure 3:**
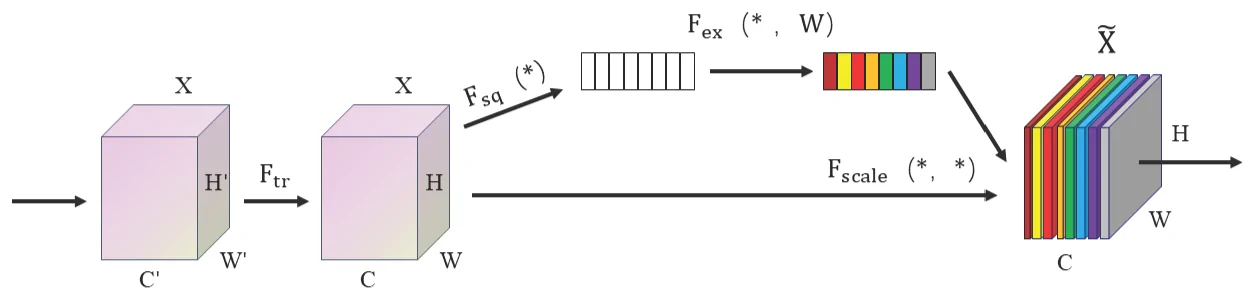
Squeeze-and-Excitation Module.

To address the problem that key behavioral features are easily overwhelmed due to the “small targets vs. large background” contradiction in the classroom scene, this paper embeds a Squeeze-and-Excitation (SE) attention module into the backbone network to achieve feature purification. This module adaptively recalibrates channel-wise feature responses by modeling interdependencies between feature channels. Its structure is shown in Figure 3.

In this paper, the SE module is chosen over other spatial attention mechanisms (e.g., CBAM) [23] based on two in-depth considerations regarding the classroom scene: (1) The priority of channel semantics. In a dense and heavily occluded classroom environment, specific behaviors (such as the vertical edges of hand-raising and the fine textures of writing) elicit distinct response patterns in the feature channels of convolutional neural networks. By compressing spatial dimensions and focusing on channel dimensions, the SE module can more effectively select these discriminative semantic channels, rather than struggling to localize in crowded space. (2) Lightweight requirement. The SE module has a concise structure and introduces only a very small number of parameters, which aligns well with the lightweight N-series configuration adopted in this paper. It achieves significant feature enhancement with almost no increase in computational cost, satisfying the efficiency demands of edge deployment.

In this paper, the SE module is placed after the MANet module. This is because MANet has already performed preliminary feature aggregation through multiple types of convolutions; introducing channel attention at this stage can precisely screen the aggregated high-order features, avoiding potential information loss caused by premature filtering after single, low-level convolution operations. In Figure 3, * is a placeholder for the input feature tensor to each operation, and colors are used solely to distinguish different channels. The operation of the SE module can be formally described as follows:

(1) Squeeze: This step applies global average pooling (GAP) to compress the spatial features (of size *H × W*) on each channel into a single global scalar, thereby aggregating global spatial context information and generating a channel descriptor vector *z* ∈ *R^C*, where *R^C* denotes the *C*-dimensional real coordinate space, and the *c*-th element is computed as:

(1) $$z_{C}=\frac{1}{H\;\times W}\sum_{i=1}^{H}\sum_{j=1}^{W}x_{c}(i,j)$$

(2) Excitation: This step aims to capture the non-linear relationships among channels and learn the weights of each channel. First, a fully connected (FC) layer (serving as a bottleneck layer with reduction ratio r) and a ReLU (Rectified Linear Unit) activation function are applied to perform a non-linear transformation on the compressed vector z. Then, another fully connected layer (restoring the original number of channels) and a Sigmoid activation function are used to finally generate a channel weight vector s∈R^C with values between 0 and 1, computed as:

(2) $$s=\sigma (W_{2}\varphi (W_{1}z))$$

where $W_{1}$ and $W_{2}$ are the weight matrices of the two fully connected layers, φ denotes the ReLU activation function, and σ denotes the Sigmoid activation function.

(3) Reweighting: Finally, the learned weight vector s is multiplied element-wise with the original feature map X channel-wise, completing the recalibration of the original features. This amplifies the feature responses of important channels while suppressing irrelevant or noisy channels.

(3) $${\tilde{X}}_{C}=s_{C}\times X_{C}$$

Through this process, the network can automatically strengthen the feature channels that are highly relevant to classroom behaviors (such as the hand region during writing and the arm posture during hand-raising), while suppressing the responses of irrelevant backgrounds (e.g., desks and walls). This feature purification mechanism provides higher signal-to-noise ratio input features for subsequent multi-scale fusion and accurate detection, serving as the first step in resolving the perception contradiction.

#### 3.3.2. Optimization of Multi-Scale Feature Fusion Mechanism

The original Hyper-YOLO adopts simple concatenation for multi-scale fusion, which cannot distinguish the importance of features at different scales. In the classroom scene where the contradiction between “details and semantics” is prominent, key small-scale details are easily diluted by large-scale semantic features.

To address the above problem, this paper introduces the bidirectional weighted feature pyramid network (BiFPN) as the core mechanism for feature fusion. Unlike fixed-weight concatenation, the core idea of BiFPN is to assign a learnable weight parameter to each input feature layer, allowing the model to determine during training whether deep semantic features or shallow detail features are more important for the final detection task. This enables the model to dynamically adjust the contribution ratio of information at different scales according to the actual content of the classroom image.

To accelerate the fusion network’s learning of small-target characteristics in the classroom scene, this paper designs a prior initialization strategy based on scale importance. We assume that features from K scales need to be fused, where a smaller index k indicates higher resolution (richer details). The initial weight vector $\omega_{init}$ is defined as:

(4) $$\omega_{init}^{\left(k\right)}=\alpha \;*\;exp\left(-\beta \;*\;\left(k-1\right)\right)+1$$

Here, α = 0.5 and β = 0.8 provide a mild scale-aware initialization that assigns P3 an initial gain of ≈1.5, reflecting the classroom prior that fine-grained behaviors (e.g., hand-raising, writing) predominantly rely on high-resolution shallow features. Since ω is fully learnable, the network autonomously redistributes weights during training. Thus, the initialization merely accelerates early-stage convergence without constraining the final data-adaptive performance.

Based on the above idea, two specific weighted fusion modules are designed to accommodate different connection scenarios:

(1) Two-branch weighted fusion (BiFPN_Concat2): This module takes two input feature maps $X_{1}$ and $X_{2}$ performs weighted fusion using two learnable weights $\omega_{1}$ and $\omega_{2}$, and its output Y is computed as follows:

(5) $$Y=\frac{ReLU(\omega_{1})\;*\;X_{1}+ReLU(\omega_{2})\;*\;X_{2}}{ReLU(\omega_{1})+ReLU(\omega_{2})+\epsilon }$$

where $X_{1}$ and $X_{2}$ are the two input feature maps to be fused, and ϵ is a small constant added for numerical stability. This computation can be regarded as a soft attention mechanism, where the network assigns higher weights to more important feature maps through learning.

(2) Three-branch weighted fusion (BiFPN_Concat3): It extends the two-branch version to three weights, $\omega_{1}$, $\omega_{2}$, and $\omega_{3}$, which are used to perform weighted summation and normalization on the three inputs $X_{1}{,X}_{2},X_{3},$ respectively.

The proposed weighted fusion mechanism does not work in isolation but closely integrates with the original hypergraph computation module of Hyper-YOLO, forming a hierarchical information processing pipeline: (1) Weighted scale fusion: BiFPN first performs adaptive weighted fusion on features of different scales, preliminarily aggregating detail and semantic information. (2) High-order semantic association: The balanced features are then fed into the hypergraph module for deep, non-local semantic relationship reasoning. (3) Enhanced information redistribution: The features enhanced by hypergraph reasoning and rich in high-order semantics are finally redistributed back to the output feature maps of each scale via the scattering mechanism for final classification and localization.

#### 3.3.3. Reconstruction of the Bounding Box Regression Loss Function

The accuracy of bounding box regression is one of the key factors determining object detection performance. The original Hyper-YOLO model adopts the CIoU loss as the optimization objective for its bounding box regression. The CIoU loss [24] extends IoU by comprehensively considering the center point distance and aspect ratio between the predicted box and the ground-truth box. Its definition is more complete than earlier IoU, GIoU, and DIoU losses [24,25]. However, in the practical application scenario of classroom behavior detection, the CIoU loss exhibits certain limitations. In the classroom environment, there exist numerous challenging samples: first, the complex background contains many occlusions, generating a large number of low-quality samples; second, the key behavioral targets are often very small, and their annotation boxes inherently suffer from biases. The geometric penalty terms in the CIoU loss impose a consistent optimization strength on all samples without the ability to distinguish sample quality, which to some extent harms the localization accuracy on high-quality samples.

To address the above problem, this paper adopts WIoU v3 as the bounding box regression loss. The core innovation of WIoU v3 lies in its dynamic non-monotonic focusing mechanism. This mechanism dynamically assigns gradient gains by evaluating the “outlierness” of anchor boxes, automatically reducing the negative impact of low-quality outlier samples while preventing simple samples from dominating the training.

The construction of WIoU v3 is based on the WIoU v1 loss, whose core formula is as follows:

(6) $$L_{WIoUv3}=r\times L_{WIoUv1}$$

where $L_{WIoUv1}=R_{WIoU}\times L_{IoU}$ is the WIoU v1 loss,

(7) $$R_{WIoU}=exp(\frac{{\left(x-x_{gt}\right)}^{2}+{\left(y-y_{gt}\right)}^{2}}{{\left(W_{g}^{2}+H_{g}^{2}\right)}^{*}})$$

is the distance attention term.

In this formula, ${\left(W_{g}^{2}+H_{g}^{2}\right)}^{*}$ indicates that this term is detached from gradient backpropagation during computation, so it is treated as a constant during training and is used only to measure “outlierness”.

r is the dynamic non-monotonic focusing coefficient, calculated as:

(8) $$r=\frac{\beta }{\delta \alpha^{\beta -\delta }}$$

$\bar{L_{IoU}}$ is the exponential moving average of $L_{IoU}$, and the momentum *m* is set as $m=1-\sqrt[{4}]{0.05}$ according to the number of training batches t and the period of accuracy improvement slowdown T *α* and *δ* are hyperparameters, which are set to 1.9 and 3.0, respectively, following the original WIoU v3 formulation. These parameters control the generic curvature and focusing point of the dynamic gradient allocation, which relies on relative outlierness *β* rather than absolute geometric thresholds. Classroom scenes naturally share the same heavy-tailed sample-quality distribution (occlusion, motion blur, annotation bias) with general detection benchmarks; consequently, the relative outlierness metric autonomously adapts to the empirical data landscape without requiring dataset-specific tuning of *α* and *δ*. Retaining the original values further ensures a fair comparison with prior studies.

The dynamic non-monotonic focusing mechanism works as follows. The outlier degree β effectively measures the quality distribution of predicted boxes. When a predicted box is a low-quality outlier sample, its β value is large and the corresponding r value decreases accordingly, thereby reducing the gradient gain for that sample and suppressing harmful gradients. For high-quality inlier samples, a small β value also leads to a small gradient gain, preventing simple samples from dominating the training. Samples of medium quality, whose β is close to 1.5, receive the largest gradient gain and become the main focus of model optimization. This dynamic adjustment mechanism enables the model to adaptively allocate attention according to the current training state, making it particularly suitable for classroom scenes where annotation quality and target difficulty vary considerably.

In this paper, WIoU v3 loss, classification loss $\left(L_{cls}\right)$ and distribution focal loss $\left(L_{dfl}\right)$ together form the overall optimization objective of the model:

(9) $$L_{total}=\lambda_{1}L_{WIoUv3}+\lambda_{2}L_{cls}+\lambda_{3}L_{dfl}$$

where $\lambda_{1}{,\lambda }_{2}{,\lambda }_{3}$ are weight coefficients balancing the contribution of each loss term.

In summary, the introduction of the WIoU v3 loss enables the model to adaptively allocate its optimization focus during training: it reduces attention to low-quality, high-uncertainty samples to prevent their unreliable gradients from interfering with the optimization direction, while moderately focusing on medium-quality samples to achieve more accurate bounding box regression. This improvement significantly enhances the model’s localization robustness and final detection accuracy in complex classroom environments, especially improving the localization stability for small-scale behavioral targets without introducing additional task-specific parameter tuning.

### 3.4. Integrated Strategy of Feature Screening, Fusion, and Optimization Across the Full Pipeline

**Figure 4:**
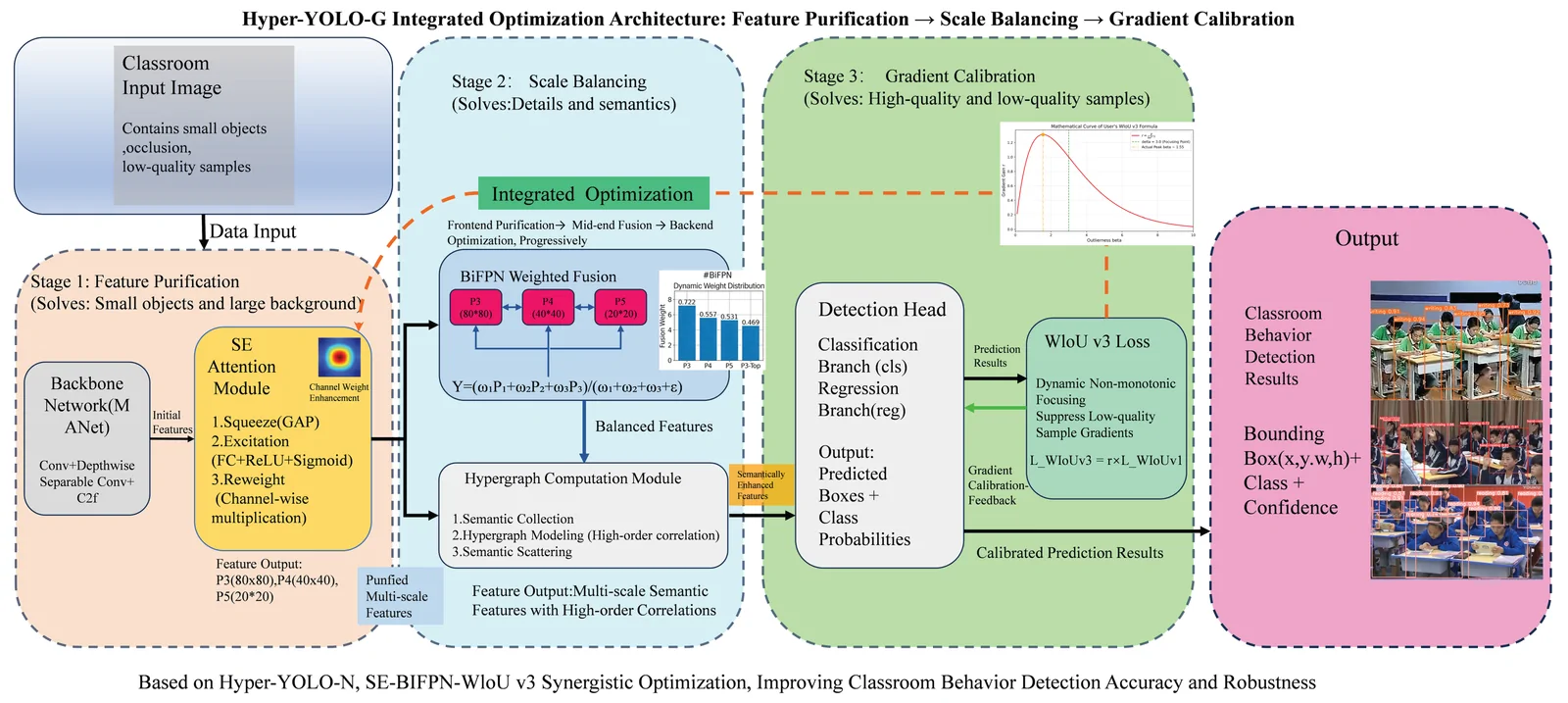
Architecture diagram of integrated optimization for classroom behavior detection in the improved model.

The proposed framework is a systematic solution tailored for complex classroom scenes. As shown in Figure 4, it is not a simple stacking of independent modules but a progressive integrated pipeline following the logic of “feature purification → scale balancing → gradient calibration”. The design principle is that each stage clears obstacles for the next, while the unified loss signal drives global joint optimization.

Interdependence of classroom contradictions. The three core challenges are structurally interdependent: the “small targets vs. large background” contradiction produces low-SNR initial features; these noisy features, if fused without selection, exacerbate the “details vs. semantics” conflict; and the resulting unstable feature distribution, combined with occlusion and blur, generates harmful gradients that aggravate the “high-quality vs. low-quality sample” problem. Isolated improvement of any single stage cannot break this chain.

Stage-wise optimization. The SE module performs feature purification by recalibrating channel responses, enhancing behavior-related channels while suppressing background interference. This delivers high-SNR feature primitives to BiFPN, preventing background noise from diluting multi-scale fusion. BiFPN then undertakes scale balancing via adaptive weighted fusion, autonomously reconciling shallow details with deep semantics. The resulting balanced feature representation stabilizes the sample distribution fed to the detection head, reducing the proportion of extreme outliers. Finally, WIoU v3 calibrates gradients through its dynamic non-monotonic focusing mechanism, suppressing harmful gradients from remaining low-quality outliers while concentrating optimization on medium-quality samples. Thus, SE alleviates the source-level perception contradiction, BiFPN resolves the transformation-level representation contradiction, and WIoU safeguards the optimization-level robustness.

End-to-end training. During standard gradient backpropagation, the localization loss from WIoU v3 propagates backward through the detection head to the neck and backbone, jointly updating the parameters of feature enhancement, fusion, and localization. This enables joint parameter updating: SE’s channel selection evolves to produce features that are more favorable for BiFPN’s weighted fusion; BiFPN’s fusion weights adjust to deliver feature distributions that stabilize WIoU’s gradient allocation; and WIoU’s loss signal indicates which channels or scales suffer from poor sample quality, implicitly guiding SE and BiFPN to focus on harder regions. The three components thereby progress toward a unified detection objective rather than optimizing isolated sub-problems.

In summary, this framework systematically overcomes the three classroom challenges through stage-wise integration: feature purification as the foundation, scale balancing as the transformation, and gradient calibration as the safeguard. The SE, BiFPN, and WIoU v3 modules complement each other to form a unified integrated optimization, collectively enhancing the baseline model into a behavior recognition system deeply adapted to complex classroom environments.

## 4. Experimental Setup and Results Analysis

### 4.1. Experimental Environment Setup

Experiments were conducted on Ubuntu 22.04.1 with Python 3.8, PyTorch 2.4.1, and CUDA 12.1. The hardware configuration includes two NVIDIA RTX 4090 GPUs with 24 GB of memory each and a 64-core Intel(R) Xeon(R) Platinum 8380 CPU.

### 4.2. Training Settings

The training settings are as follows: learning rate: The initial learning rate is set to 0.0175. The first five epochs serve as a warm-up phase, during which the learning rate gradually increases from the initial value to ensure stability. After warm-up, the learning rate decays to 0.0012, promoting stable and effective convergence.

Optimizer: SGD with a momentum of 0.98 and a weight decay of 0.00025. SGD is chosen for its stable gradient updates. Batch size: 16, ensuring efficient GPU memory utilization and stable model updates. Number of epochs: The model is trained for up to 300 epochs, with early stopping patience of 15 epochs. Input resolution is 640 × 640, with loss weights and augmentations following the Ultralytics default configuration; source code will be released upon acceptance.

### 4.3. Experimental Analysis

#### 4.3.1. Comparative Experiments of Algorithms on the Dataset

This study uses the public 4.2k HRW YOLO Dataset [26] for model training and testing. Released by Chengdu Neusoft University, this dataset contains 4366 real classroom images annotated with three behaviors: hand-raising (10,078 instances), reading (5882), and writing (2539). The dataset covers various lighting conditions, viewing angles, and different degrees of occlusion, and thus has good challenge and representativeness. The evaluated algorithm models include YOLOv5n, YOLOv8n, YOLOv11n, and Hyper-YOLOn. Performance is evaluated using Precision, Recall, mAP@50, and mAP@50:95. Among them, mAP@50 is the core metric for evaluating detection accuracy. All experiments were conducted under identical settings to ensure comparability. The experimental results of different models are as follows:

As shown in Table 1, Hyper-YOLO-G achieves 73.7% mAP@0.5, a 4.9% improvement over the baseline Hyper-YOLOn, validating the overall effectiveness of the integrated framework. Precision reaches 0.674, indicating higher accuracy in identifying positive samples and a lower false positive rate. This is mainly attributed to the ‘feature purification’ achieved by the SE module, which effectively suppresses background interference and enhances prediction confidence. Recall increases to 0.694 (3.7% higher than baseline), demonstrating that the model significantly improves coverage of positive samples, particularly small and occluded targets. The “scale balancing” provided by the BiFPN module ensures sustained perception of multi-scale targets, especially small ones. Overall, the proposed model achieves a better balance between accuracy and robustness.

#### 4.3.2. Comparative Experiments on the Student Behaviour Detection.v6i.yolov8 Dataset

To evaluate generalization and scene adaptability, comparative experiments were further conducted on the public ‘Student Behaviour Detection.v6i.yolov8’ dataset [27]. Collected from real classrooms, this dataset contains 4065 images with 153,762 annotated instances across 12 behavior categories, including head down, sitting straight, bending, turning head, looking up, holding a book, reading, writing, using a mobile phone, sleeping, and hand-raising. Compared with the 4.2k HRW YOLO dataset, it features richer categories, more diverse scenes, and more pronounced inter-class similarity and intra-class variability, posing greater challenges to semantic discrimination and multi-scale adaptation.

Notably, the baseline Hyper-YOLO achieves 73.3% mAP@0.5 in Table 2, which is higher than the 68.8% in Table 1. This difference stems from inherent dataset characteristics: the 4.2k HRW YOLO dataset focuses on challenging small-scale fine actions such as hand-raising and writing, whereas the Student Behaviour Detection dataset includes relatively easy large-scale postures such as holding a book and sitting straight. The overall task difficulty of the latter is different, leading to the discrepancy in the absolute performance of the baseline model.

However, the core focus of this study lies in the improvement margin and trend relative to the baseline. As shown in Table 2, Hyper-YOLO-G still achieves comprehensive gains (4.9% mAP@0.5 improvement) under this more challenging multi-category scenario, with concurrent increases in precision and recall. This strongly confirms that the proposed framework is not over-fitted to a specific dataset and that its mechanisms for addressing core classroom challenges—small targets, multi-scale variation, and occlusion—generalize well.

**Figure 5:**
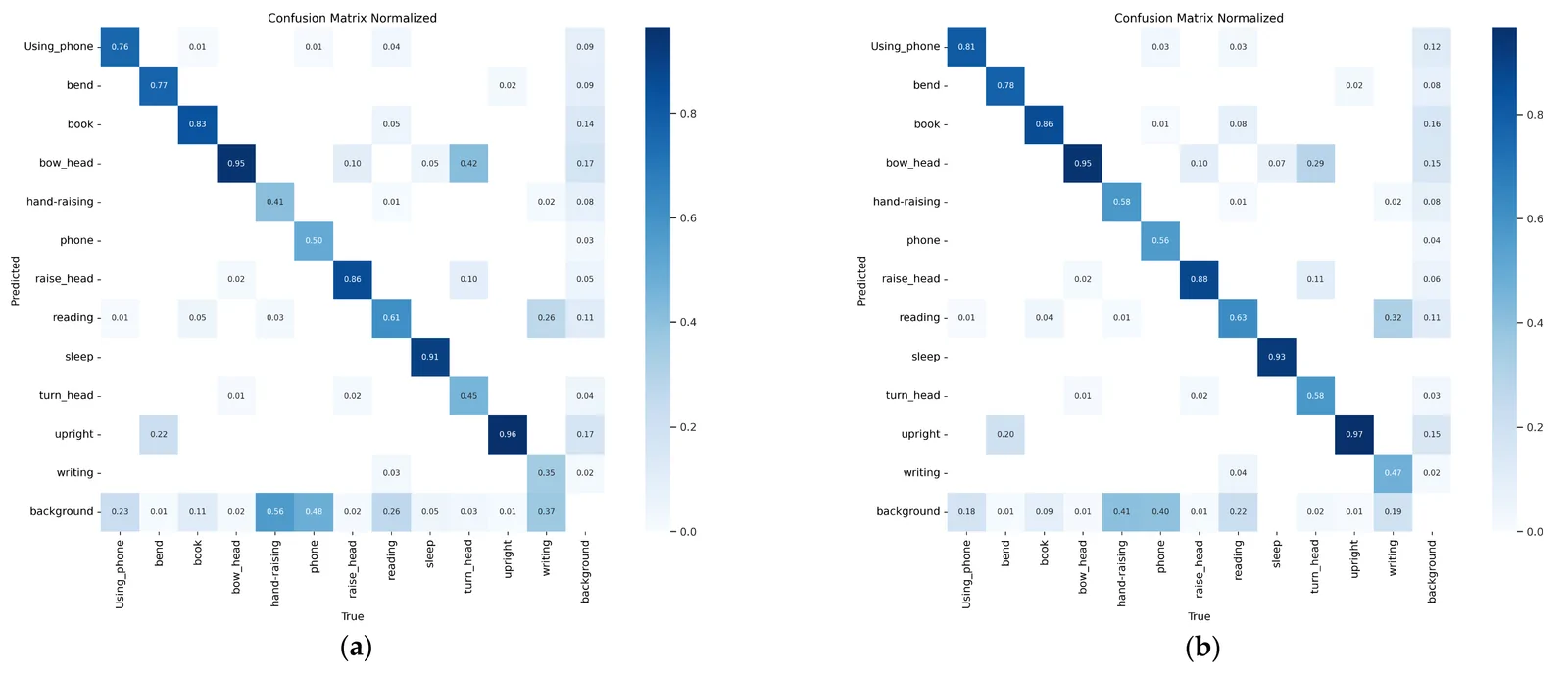
Normalized confusion matrices. (**a**) Original Hyper-YOLO; (**b**) Improved Hyper-YOLO-G.

To further analyze the model’s performance across various behavior categories, Figure 5 presents the normalized confusion matrices of the original Hyper-YOLO and our improved Hyper-YOLO-G on the test set, where Figure 5a corresponds to the baseline model and Figure 5b corresponds to the proposed model. Comparative observation shows that the improved model obtains higher recognition accuracy for most student behavior classes and alleviates inter-class misclassification significantly. This phenomenon illustrates that our framework boosts not only the target localization recall but also the semantic distinguishability among different behaviors via cleaner and more balanced feature representations. Moreover, it verifies that the proposed integrated framework is not an overfitted optimization tailored to a single dataset. Its core design concepts, including feature purification, multi-scale balance and gradient calibration, possess strong generalization capability for complex and diverse classroom behavioral detection tasks.

In summary, even on the challenging multi-class student behavior dataset, our improved model achieves favorable generalization performance and consistent accuracy gains, which validates its robust practicability and application potential in real classroom education scenarios.

### 4.4. Ablation Experiments and Attention Mechanism Selection

#### 4.4.1. Ablation Analysis of Proposed Components

To systematically verify the effectiveness of SE, BiFPN, and WIoU v3, ablation experiments were conducted on the 4.2k HRW YOLO dataset using controlled variables. The experiments are divided into two stages: (1) independent module verification on the original Hyper-YOLO backbone; (2) incremental integration on the lightweight Hyper-YOLO-Light backbone. We add each component incrementally and evaluate all schemes based on detection accuracy and model complexity. The results are listed in Table 3.

From the analysis of Table 3, the following conclusions can be drawn:

Independent effectiveness of each module: Simplifying the detection head cuts parameters by ~0.31 M and lifts mAP@50 slightly to 0.693. Adding SE to the original full head boosts mAP@50 to 0.710, a 2.0% improvement over the baseline. Individually inserting BiFPN or WIoU v3 brings 1.7% (0.707 ± 0.0025) and 1.3% (0.703 ± 0.003) mAP@50 gains, respectively, with barely extra parameters. Small standard deviations across three repeated training runs prove stable performance gains from the core modules. All proposed modules raise detection accuracy without obvious complexity growth.

Combined effect among modules: The combination of simplified head and SE only achieves mAP@50 = 0.704, inferior to the full-head SE model. With fewer MANet layers, the lightweight backbone weakens feature representation and weakens SE’s benefits, yet this group still surpasses the baseline by 1.4% with fewer parameters. Integrating a simplified head, SE and BiFPN obtains mAP@50 = 0.726. Though its total gain does not exceed the sum of separate module improvements, this triple combination achieves remarkable cumulative improvement through sequential feature enhancement. In addition, BiFPN slightly reduces mAP@50:95 to 0.507, which can be fully recovered and further elevated by WIoU v3.

Stable gain from the loss function: Equipping the three-module model with WIoU v3 forms our final model. Its mAP@50 rises from 0.726 to 0.740 ± 0.003 and mAP@50-95 from 0.507 to 0.523 ± 0.002, gaining 5.0% mAP@50 against the baseline (0.690 ± 0.002). The dynamic focusing mechanism of WIoU v3 complements the foregoing feature modules and improves detection performance on complex classroom scenes.

Metrics of the baseline, BiFPN-only, WIoU v3-only and final models are averaged over three runs with seeds 1, 42 and 2025 and reported as mean ± standard deviation. All other ablation groups use single-run results.

#### 4.4.2. Comparison of Attention Mechanisms

To select a suitable attention module, we compare SE, CBAM, and CA (Coordinate Attention) on vanilla Hyper-YOLO with the complete detection head. All experiments strictly follow the single-variable principle. The quantitative results are listed in Table 4.

As shown in Table 4, the SE module achieves the optimal mAP@50 and mAP@50:95. Although CBAM and CA achieve slightly higher precision, their recall and overall detection accuracy are inferior to SE. Moreover, SE introduces fewer additional parameters.

### 4.5. Comparison and Analysis with Mainstream State-of-the-Art Detection Models

To validate the performance superiority of Hyper-YOLO-G in complex classroom scenes, we compare it with two representative recent SOTA detectors: Gold-YOLO [28] and YOLO-MS [29]. Gold-YOLO relies on a gather-distribute global aggregation scheme, while YOLO-MS focuses on multi-scale screening optimization; both are typical mainstream improvements for general object detection. All models are trained and tested under identical configurations on the 4.2k HRW YOLO dataset, with full quantitative results listed in Table 5.

As shown in Table 5, Hyper-YOLO-G achieves 0.737 mAP@50, surpassing Gold-YOLO (0.667) and YOLO-MS (0.690) by 7.0% and 4.7%, respectively. It also obtains the highest mAP@50:95 among all competitors, demonstrating stronger high-precision localization capacity. The domain-adaptive “feature purification—scale balancing—gradient calibration” integrated pipeline proposed in this paper outperforms generic multi-scale and global aggregation designs for classroom behavior recognition.

Class-wise results further reveal the advantages of our framework:

1. Fine-grained writing targets: Gold-YOLO’s global aggregation easily blurs tiny hand and pen features, yielding only 0.645 mAP@50 for writing. Benefiting from SE channel screening and BiFPN’s weighted retention of shallow fine features, our method reaches 0.724 mAP@50 for this category, effectively solving small-target feature attenuation.
2. Occluded raising-hand targets: YOLO-MS adopts multi-scale selection but uses uniform geometric loss, which is vulnerable to misleading gradients from occluded blurry samples. Equipped with WIoU v3’s dynamic non-monotonic focusing, our model suppresses interference from low-quality samples and lifts hand-raising mAP@50 to 0.817, far exceeding YOLO-MS’s 0.790.

In summary, unlike simple multi-scale or global information enhancement, Hyper-YOLO-G’s progressive integrated design specifically addresses the two core pain points of classroom detection—faint small-target features and noisy occluded samples—delivering comprehensive gains over mainstream SOTA methods.

### 4.6. Visualization

#### 4.6.1. Visualization of Detection Performance

To intuitively verify perception ability and robustness, this section selects typical test samples under varying lighting, crowding, and shooting angles for visual comparison. As shown in Table 6, we compare the original input images, the detection results of the baseline model, and those of the improved model. Through qualitative analysis, the improved model demonstrates significant advantages in the following four key dimensions:

1. Improved recognition of small and occluded targets: As shown in the second row of Table 6, the improved model effectively recognizes targets with small pixel sizes or partial occlusion. This model significantly reduces the missed detections commonly observed in the baseline model, thereby substantially improving the overall recall.
2. Optimized localization accuracy: As shown in the third row of Table 6, the improved model produces more compact and precise bounding boxes, tightly fitting target contours and reducing boundary redundancy. This reflects that the model is more stable in the bounding box regression task, ensuring high localization accuracy of the model outputs, which is crucial for subsequent behavior statistics and analysis.
3. Enhanced prediction confidence: As shown in the fourth row of Table 6, the improved model achieves consistently higher confidence scores across all successfully detected targets. Higher confidence means that the improved model has stronger discriminative power for features, effectively suppressing noise and background interference that may be introduced during detection, thus making the model more robust in real complex classroom environments.
4. Maintained discriminative ability for different behavioral features: As shown in the fifth row of Table 6, the improved model corrects baseline errors such as misclassifying writing as reading. The improved model exhibits stronger recognition accuracy than the baseline model in such scenarios, demonstrating that the optimizations in this work not only enhance target localization ability but also effectively strengthen the model’s semantic discrimination of different behavior categories.

Summary: Based on the above visual comparison, Hyper-YOLO-G outperforms the baseline in precision, recall, and robustness, proving its effectiveness in complex classroom behavior recognition.

#### 4.6.2. Visualization of Core Mechanism Effectiveness

This section reveals the working principles of SE, BiFPN, and WIoU v3 through mechanism visualizations, validating the rationality of the proposed framework improvements.

**Figure 6:**
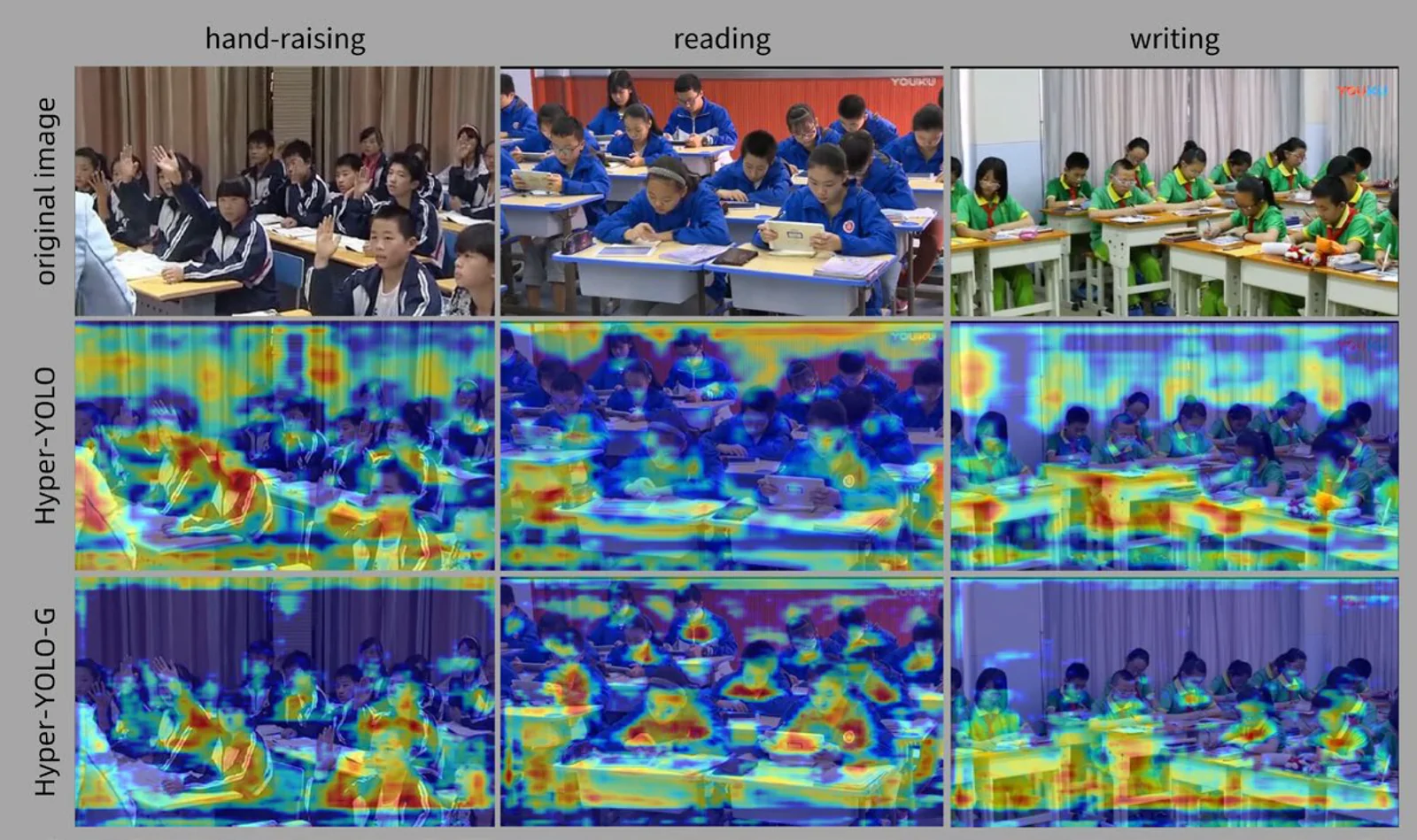
Heatmap visualization of Different Algorithms.

Figure 6 shows the original images of three classroom behaviors (“hand-raising”, “reading”, and “writing”) together with the attention heatmaps of Hyper-YOLO and Hyper-YOLO-G. Compared with the baseline, Hyper-YOLO-G with the embedded SE module automatically strengthens key behavioral feature channels while suppressing irrelevant background interference. Whether for hand-raising postures, reading interactions between book and face, or writing hand movements, Hyper-YOLO-G’s attention focuses more accurately on these core behavioral regions rather than dispersing over irrelevant backgrounds such as desks and curtains. This characteristic corresponds to the improvement in detection performance, fully demonstrating the effectiveness of the SE module in enhancing the discriminability of key behavioral features and reducing background interference. Together with the BiFPN multi-scale fusion mechanism, it collectively improves the accuracy of behavior detection in complex classroom scenes.

**Figure 7:**
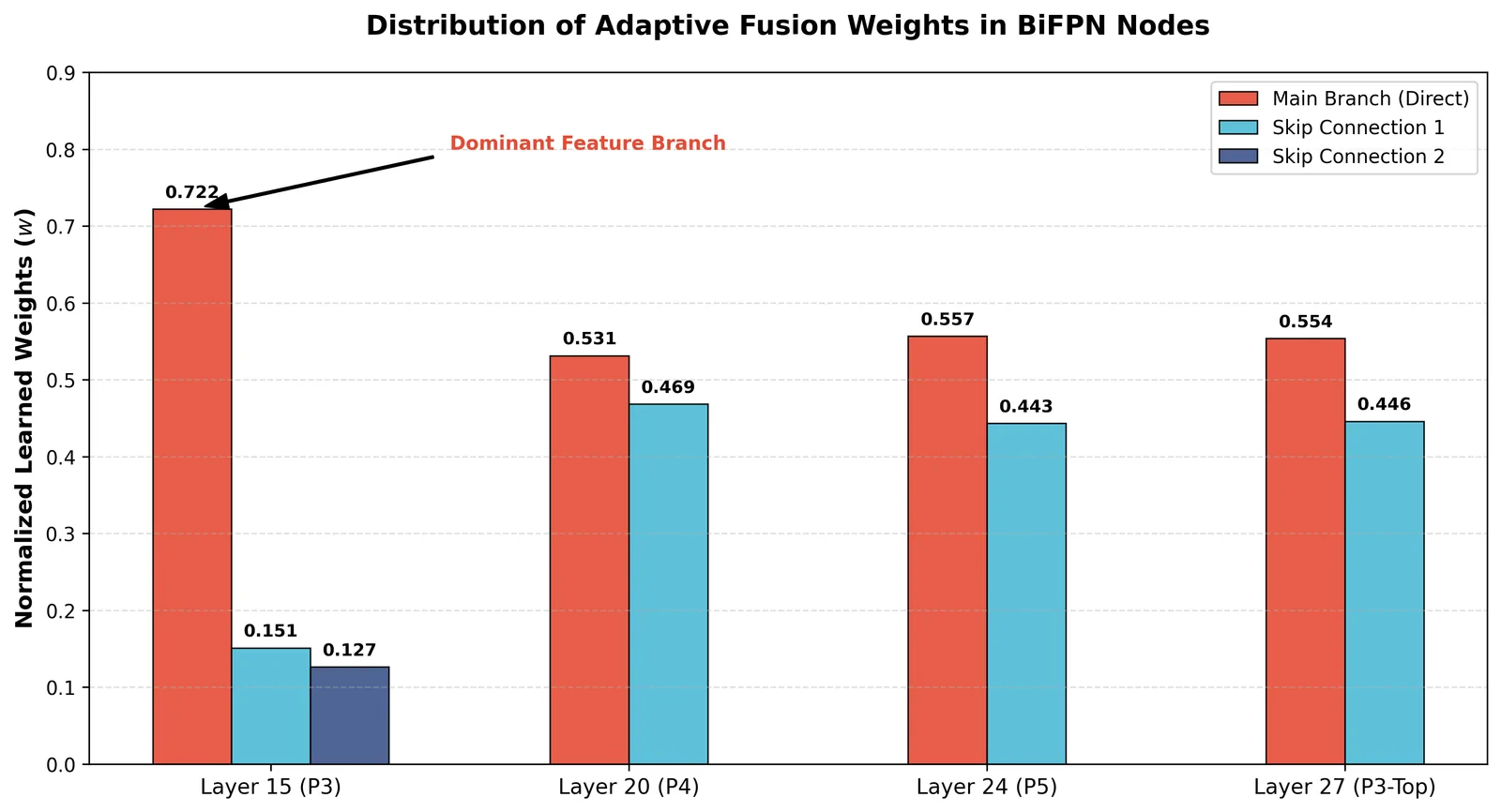
Distribution of Adaptive Fusion Weights in Nodes.

Figure 7 presents the adaptive fusion weight distribution of BiFPN nodes at different feature layers, intuitively reflecting its dynamic weighting strategy for multi-scale features. At the shallow P3 layer (corresponding to small-scale detail features, such as hand movements and writing tools in classroom behaviors), the weight of the direct branch reaches 0.722, becoming the “dominant feature branch”. This design enhances the contribution of small-scale detail features and prevents them from being diluted by deep semantic features during fusion. As the feature layer progresses to P4 and P5 (corresponding to medium- and large-scale target features), the weights of the direct branch and the skip-connection branch gradually become balanced, reflecting the adaptive integration capability of BiFPN for features at different scales. Even at the P3-Top layer, the direct branch still maintains a relatively high weight proportion, further ensuring the effective transmission of small-scale behavioral features.

**Figure 8:**
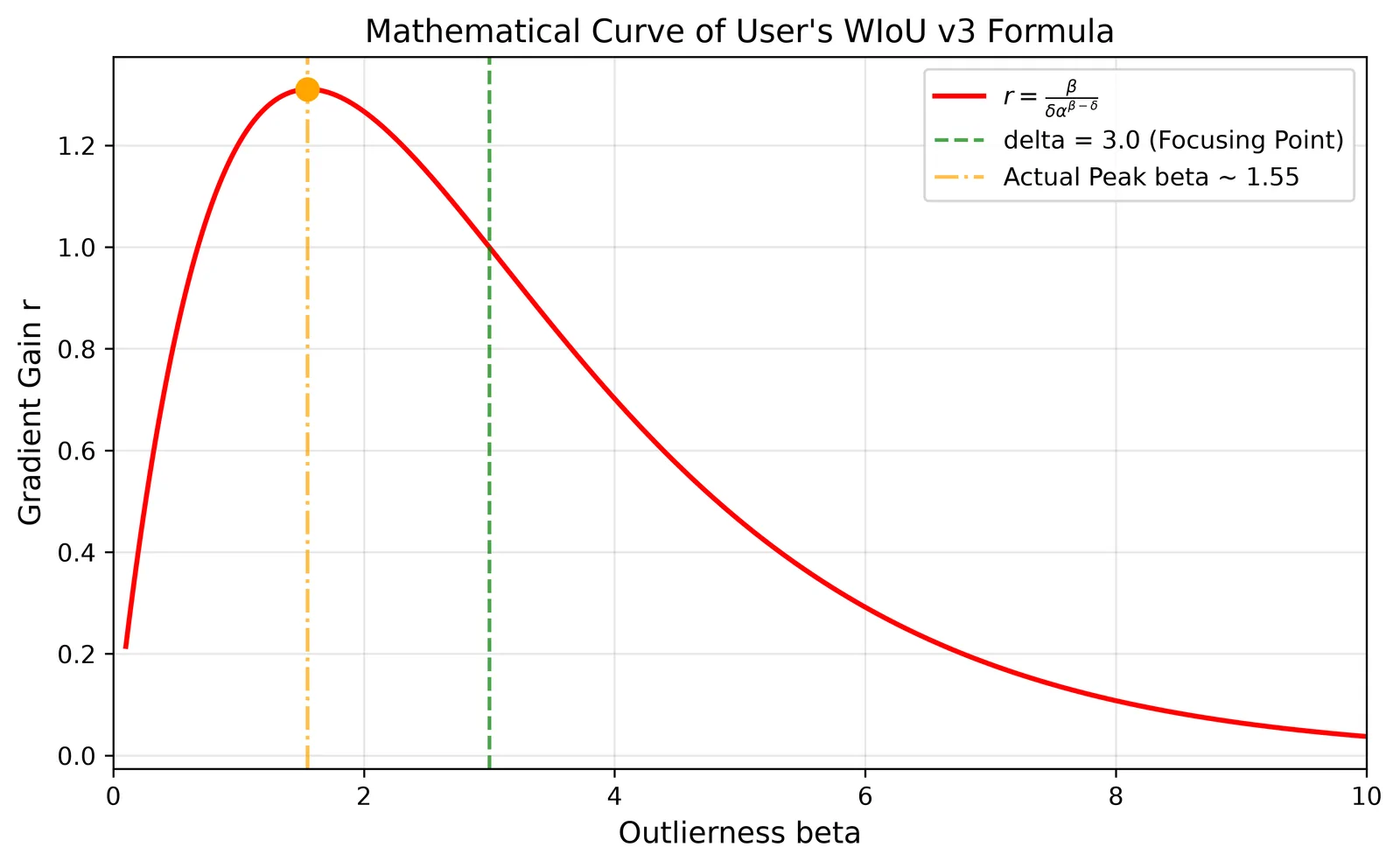
Gradient gain curve of WIoU v3.

To verify the optimization capability of WIoU v3, we plot its gradient gain against sample outlierness β in Figure 8. The curve exhibits a non-monotonic unimodal shape: both high-quality inliers (small β) and low-quality outliers (large β) are assigned reduced gradient gains, whereas medium-quality samples near β ≈ 1.5 receive the strongest signals. This prevents easy samples from dominating training while suppressing harmful gradients from noisy outliers, ensuring robust localization in complex classroom scenes.

#### 4.6.3. Failure Case Analysis

Although Hyper-YOLO-G achieves strong performance, occasional failures still occur under challenging conditions (Table 7). Most errors stem from severe occlusion, dense scenes with small targets, and image blur, which degrade discriminative features or increase localization ambiguity. These cases are confined to extreme environments, while the proposed method still outperforms existing lightweight detectors overall. Future work will improve robustness through richer contextual cues and enhanced feature representation for small and occluded targets.

## 5. Conclusions and Outlook

### 5.1. Research Summary

Targeting the three contradictions in classroom student behavior recognition, this paper proposes a scene-driven progressive integrated optimization framework, Hyper-YOLO-G. Through a progressive integrated design of feature purification (SE), scale balancing (BiFPN), and gradient calibration (WIoU v3), the framework systematically enhances the basic perception and optimization capabilities of Hyper-YOLO. Experiments on public datasets validate the effectiveness and generalization of the framework, providing a lightweight solution for real-time behavior analysis in complex educational scenarios.

### 5.2. Future Outlook

Although the proposed method achieves significant improvements, several limitations remain. First, the model only relies on single frames and does not exploit the temporal dynamic information in classroom behaviors, limiting its ability to recognize context-dependent actions such as “discussing” and “answering questions”. Second, under extreme occlusion or very low illumination, the miss rate remains high. Moreover, the current dataset size (about 4000 images) and category diversity (only three main behaviors) constrain the generalization boundary of the model.

Future work will focus on three aspects: (1) incorporating video temporal information (e.g., 3D convolutions or temporal attention) to capture behavioral dynamics; (2) introducing human pose estimation or part detection as auxiliary branches to enhance robustness against local occlusion; (3) constructing a larger-scale, multi-category classroom behavior dataset with temporal annotations, and exploring multi-modal (e.g., audio, text) fusion strategies.

## Figures and Tables

**Table 1:** Test results of different models.

Model	Precision	Recall	mAP@50	mAP@50:95
YOLOv5n	0.626	0.652	0.674	0.468
YOLOv8n	0.608	0.670	0.679	0.475
YOLO11n	0.612	0.668	0.679	0.474
Hyper-YOLOn	0.645	0.657	0.688	0.489
Hyper-YOLO-G	0.674	0.694	0.737	0.521

**Table 2:** Performance on the Student Behaviour Detection.v6i.yolov8 Dataset.

Model	Precision	Recall	mAP@50	mAP@50:95
Hyper-YOLOn	0.752	0.704	0.733	0.498
Hyper-YOLO-G	0.779	0.740	0.782	0.528

**Table 3:** Comparative Analysis of Different Modules on Hyper-YOLO Performance.

Hyper-YOLO	Hyper-YOLO-Light	SE	BiFPN	WIOU	mAP@50	mAP@50:95	Parameters	GFLOPs
√					0.690 ± 0.002	0.489 ± 0.001	3,942,649	10.8
	√				0.693	0.495	3,633,913	10.0
√		√			0.710	0.512	3,950,841	10.8
√			√		0.707 ± 0.0025	0.509 ± 0.0017	3,942,659	10.8
√				√	0.703 ± 0.003	0.505 ± 0.003	3,942,649	10.8
	√	√			0.704	0.509	3,634,937	10.0
	√	√	√		0.726	0.507	3,647,362	10.1
	√	√	√	√	0.740 ± 0.003	0.523 ± 0.002	3,647,362	10.1

**Table 4:** Comparison of different attention mechanisms.

Attention Model	Precision	Recall	mAP@50	mAP@50:95	Parameters
SE	0.65	0.667	0.71	0.512	3,950,841
CBAM	0.668	0.658	0.7	0.493	4,008,539
CA	0.674	0.651	0.704	0.507	3,955,497

**Table 5:** Performance Comparison Between the Proposed Method and SOTA Models on the Classroom Behavior Dataset.

Model	Class	Precision	Recall	mAP@50	mAP@50:95
Gold-YOLO	All	0.67	0.645	0.667	0.449
	hand-raising	0.71	0.729	0.764	0.504
	reading	0.66	0.592	0.593	0.399
	writing	0.56	0.607	0.645	0.445
YOLO-MS	All	0.641	0.680	0.690	0.443
	hand-raising	0.736	0.760	0.790	0.507
	reading	0.567	0.620	0.622	0.381
	writing	0.621	0.660	0.659	0.442
Ours	All	0.674	0.694	0.737	0.521
	hand-raising	0.766	0.772	0.817	0.572
	reading	0.611	0.641	0.669	0.464
	writing	0.646	0.668	0.724	0.526

**Table 6:** Comparison of Detection Performance: Original Model vs. Improved Model.

	Original Image	Original Model	Improved Model
Comparison of Missed Detection Cases of the Improved Model in Complex Classroom Scenarios	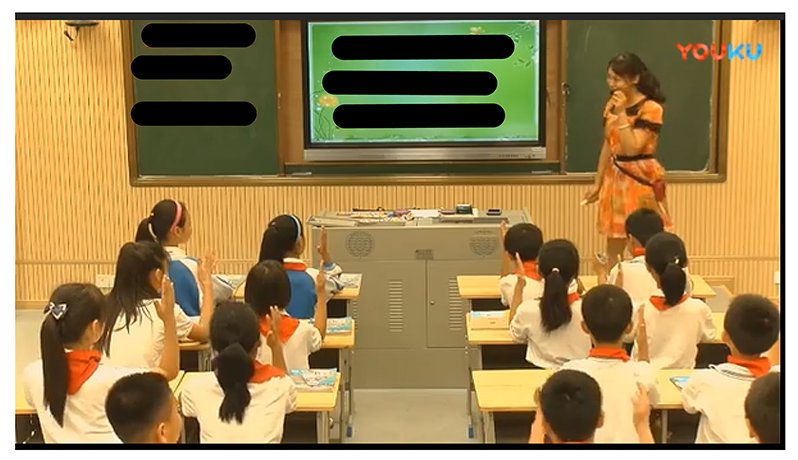	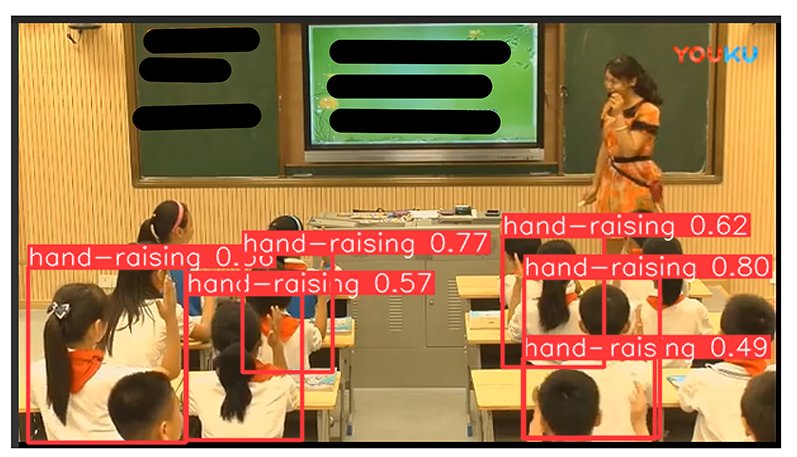	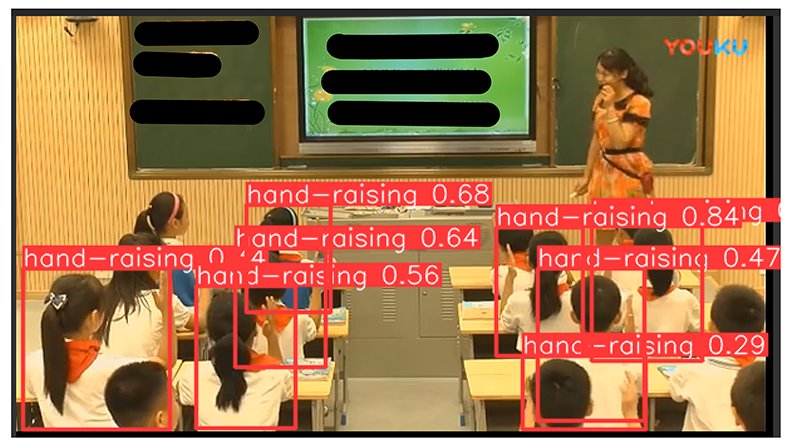
Comparison of Bounding Box Localization Accuracy Between the Improved Model and the Baseline Model	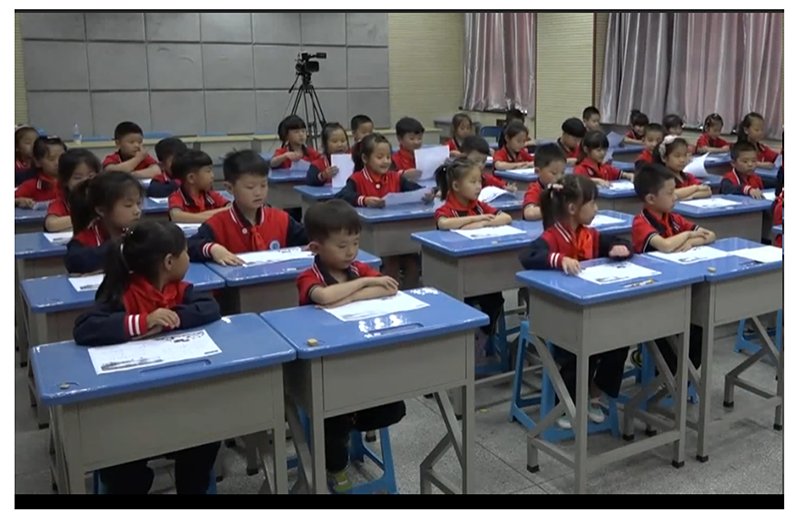	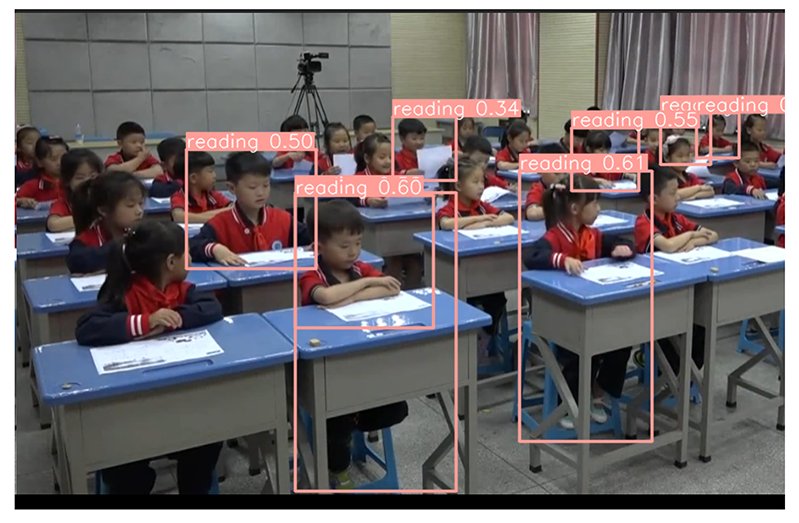	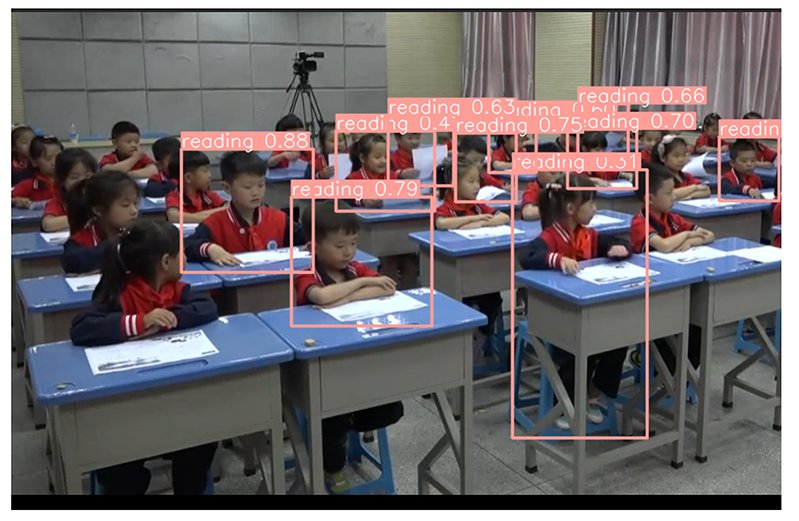
Comparison Examples of Prediction Confidence Among Different Models	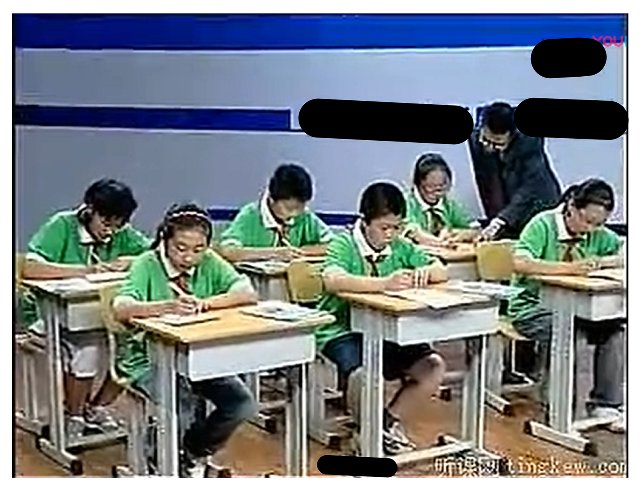	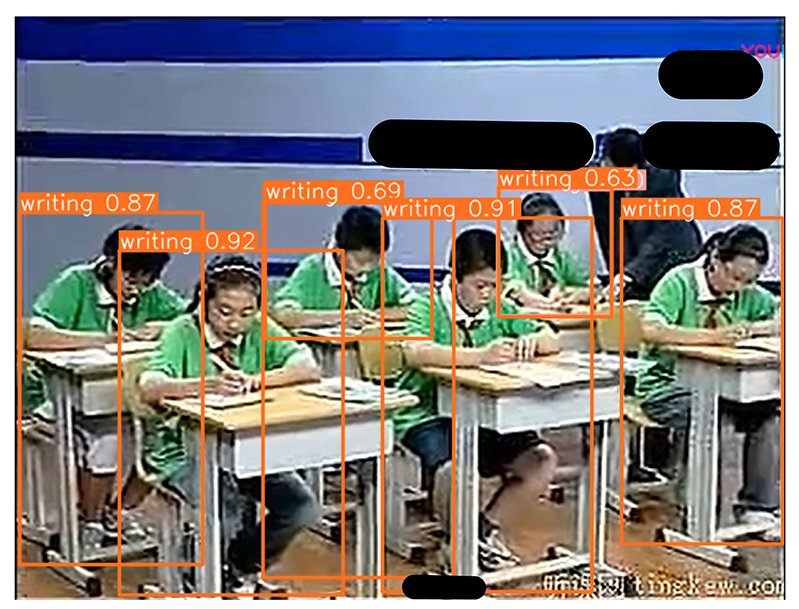	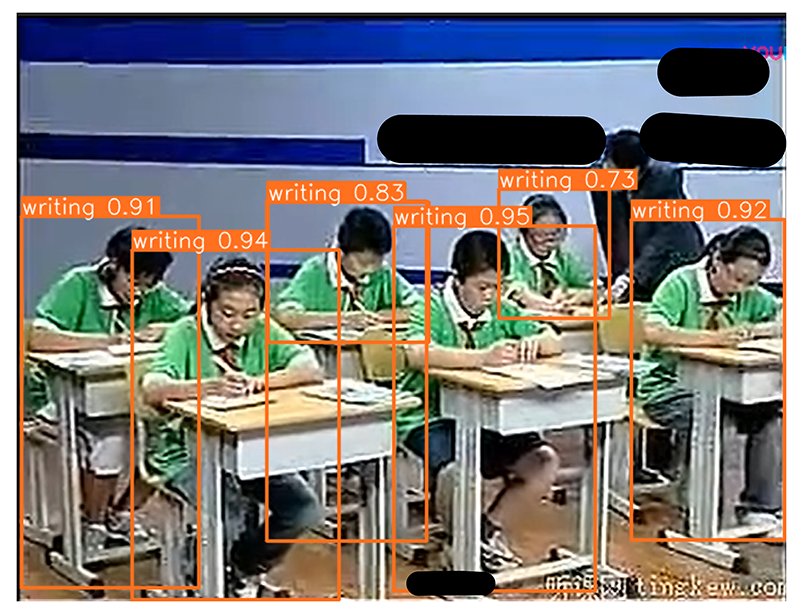
Example of Recognition Correction for Semantically Similar Behavior Categories	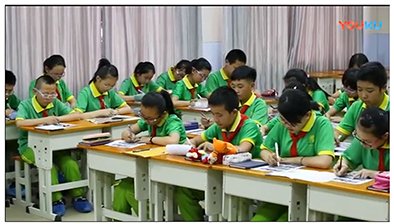	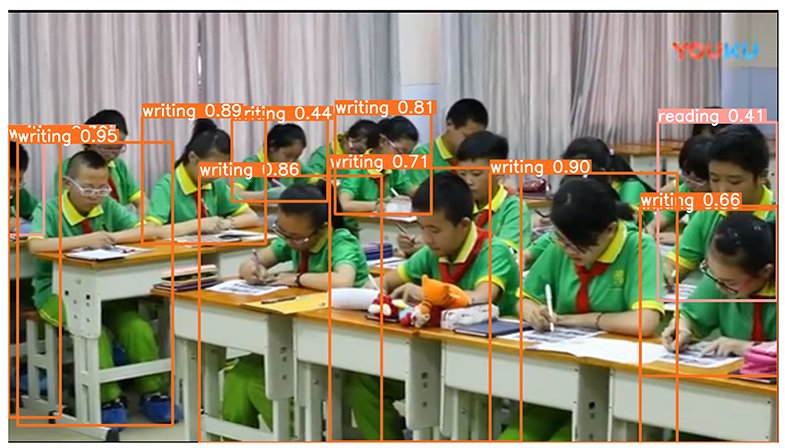	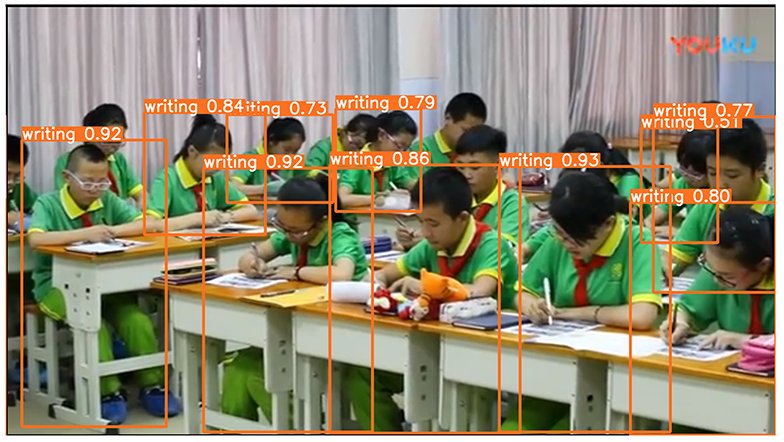

**Table 7:** Typical failure cases and root cause analysis of Hyper-YOLO-G.

Error Scenario & Phenomenon	Ground Truth	Prediction	Root Cause Analysis
Severe occlusion: Front-row students occlude >50% of the target, causing the “raise-hand” instance to be missed.	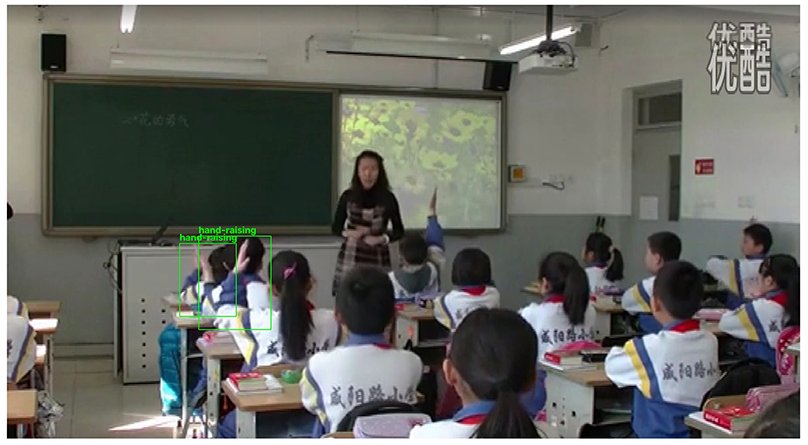	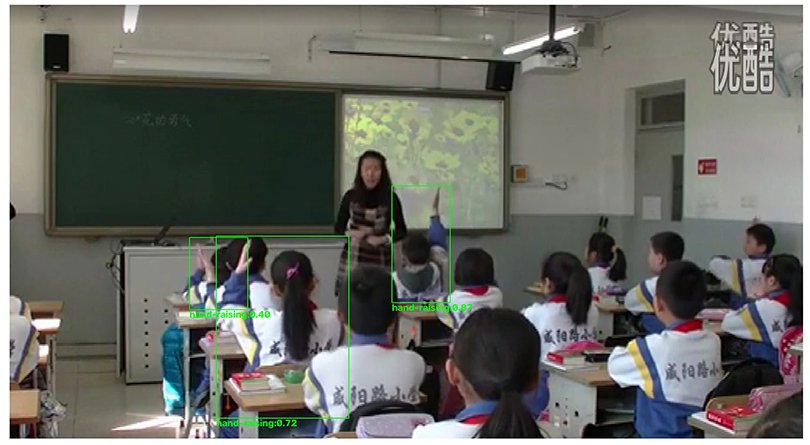	Discriminative feature loss: Visual cues of the target are fully occluded, making single-frame information inadequate for identification.
Dense small targets: Back-row low-resolution instances are misclassified.	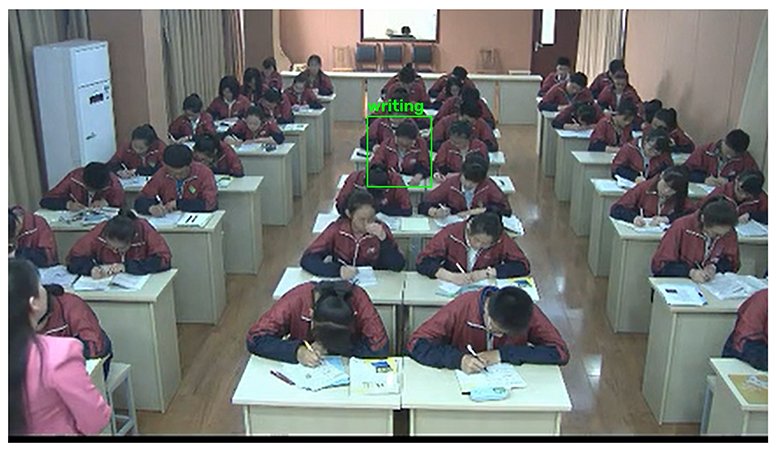	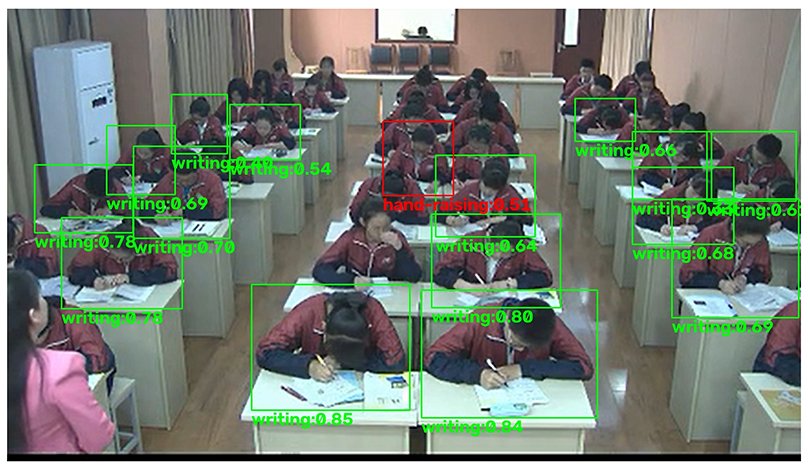	Insufficient scale resolution: Partial occlusion of small targets causes confusion in shallow-scale features, leading to misclassification of behavioral categories.
Image blur: Severe blur causes the “hand-raising” target to be missed.	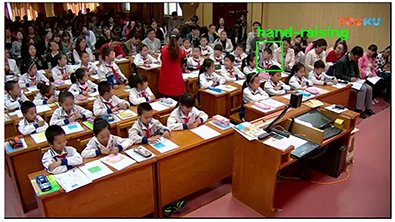	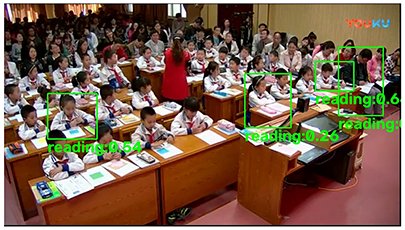	Texture detail attenuation: Failure to extract fine-grained target details results in misclassification.

## Data Availability

The datasets generated during and/or analyzed during the current study are available from the corresponding authors upon reasonable request.
